# Surgical management of pancreatic neck cancer: an ongoing dilemma

**DOI:** 10.1007/s13304-025-02453-1

**Published:** 2025-11-13

**Authors:** Davide De Sio, Claudio Fiorillo, Lodovica Langellotti, Beatrice Biffoni, Chiara Lucinato, Fausto Rosa, Vincenzo Tondolo, Giuseppe Quero, Sergio Alfieri, Roberta Menghi

**Affiliations:** 1 Pancreatic Surgery Unit, Department of Surgery, Gemelli Pancreatic Center, CRMPG (AdvancedPancreatic Research Center), Fondazione Policlinico Universitario “Agostino Gemelli” IRCCS, Largo Agostino Gemelli, 8, 00168 Rome, Italy; 2 Università Cattolica del Sacro Cuore Di Roma, Largo Francesco Vito 1, 00168 Rome, Italy; 3 General Surgery Unit, Fatebenefratelli Isola Tiberina – Gemelli Isola, Via di Ponte Quattro Capi, 39, 00186 Rome, Italy

## Abstract

The surgical management of pancreatic neck adenocarcinoma (neck PDAC) ranges from extended pancreaticoduodenectomy (ePD) to subtotal left pancreatectomy (sLP) and total pancreatectomy, with no clear consensus on the optimal approach. This study aimed to compare ePD and sLP in terms of perioperative and long-term outcomes. All patients who underwent ePD or sLP for neck PDAC were retrospectively reviewed and compared for perioperative and long-term outcomes. Forty-six patients were included: 18 (39.1%) underwent ePD and 28 (60.9%) sLP. ePD was associated with a higher rate of neoadjuvant treatment, longer operative time, and longer hospital stay. The most frequently involved lymph-node stations were #13–17 in the ePD group (44.4%) and #18 in the sLP group (42.9%). Overall survival (OS) was similar (p = 0.06), while disease-free survival (DFS) was longer in the sLP group (16 [9–22] vs 12 [5–18] months in the ePD group; p = 0.03). Due to the high rate of nodal metastases in station #13–17 and #18, both procedures may be inadequate as surgical treatment of neck PDAC. While sLP appears to offer better long-term outcomes, the heterogeneity of the study population limits generalizability. Larger prospective studies are needed to determine the most effective surgical approach.

## Introduction

The incidence rate of pancreatic ductal adenocarcinoma (PDAC) has significantly raised over the past decades and currently represents the fourth leading cause of cancer-related death[1, 2]. Surgical resection remains the only potentially curative treatment, although only 20% of patients are eligible for surgery at the time of diagnosis[3]. The choice of surgical procedure for PDAC strictly depends on tumor location. While pancreaticoduodenectomy (PD) and distal pancreatectomy (DP) are recognized as the standard surgical treatments for PDAC of the pancreatic head and body/tail, respectively, there is currently no consensus regarding the optimal surgical approach for tumors located in the pancreatic neck. Pancreatic neck represents a border area between the pancreatic body and head, located medially to the gastroduodenal artery (GDA), anterior to the portal vein (PV) and caudal to the common hepatic artery (CHA) [4–6]. These anatomical characteristics make tumors of the pancreatic neck a distinct clinical entity compared to tumors of the head and body-tail, with a hypothetical different biological behavior, particularly with respect to lymphatic drainage[4, 7, 8] . In this context, only limited and inconclusive evidence is currently available in the literature regarding the optimal surgical approach for pancreatic neck PDACs. As a result, most surgeons tend to treat them similarly to tumors of the head or body-tail. Conversely, some authors have proposed alternative or more extensive surgical procedures, such as extended pancreatoduodenectomy (ePD), central pancreatectomy (CP), total pancreatectomy (TP) or subtotal left pancreatectomy (sLP) [4, 5, 9, 10], to achieve better oncological outcomes in this subset of patients. However, only a few studies with conflicting data have assessed the potential long-term benefits of one procedure over another [4, 5, 7, 9, 10]. This is mainly due to the rarity of tumors located in the pancreatic neck, which results in small comparative cohorts and limits the ability to draw robust conclusions. Additionally, most studies have short follow-up periods. The lack of a uniform definition for the pancreatic neck further introduces bias in surgical treatment selection. Lastly, the retrospective design of the majority of available studies poses another limitation in objectively identifying the most appropriate surgical strategy.

Based on these premises, the aim of this study was to compare the short- and long-term outcomes, namely overall (OS) and disease-free survival (DFS), between ePD and sLP in patients with pancreatic neck PDAC treated at a tertiary referral center for the surgical treatment of pancreatic tumors.

## Material and methods

### Patient selection and data collection

After Institutional Review Board (IRB) approval, only patients who underwent ePD or sLP for pancreatic neck PDAC between January 2017 and December 2023 at the Pancreatic Surgery Unit of the Fondazione Policlinico “Agostino Gemelli” IRCCS of Rome were retrospectively included in the study. Pancreatic neck PDACs were defined as tumors arising within the 2-cm pancreatic segment located between the right and left border of the PV/superior mesenteric vein (SMV) without involvement of the GDA. All cases were routinely discussed at the multidisciplinary tumor board after diagnosis [11]. Neoadjuvant treatment (NAT) was recommended in cases of borderline resectable or locally advanced disease, with re-evaluation following completion of the scheduled regimen.

Data were retrospectively extracted from prospectively maintained institutional databases. Clinico-demographic variables included sex, age, body mass index (BMI) and American Society of Anesthesiologists (ASA) score. Perioperative variables collected included NAT administration, operative time, vascular resection, post-operative complications and length of hospital stay (LOS). Post-operative complications were classified according to the Clavien-Dindo classification [12]. Post-operative pancreatic fistula (POPF), delayed gastric emptying (DGE) and post-pancreatectomy hemorrhage (PPH) were defined and graded according to the International Study Group of Pancreatic Surgery (ISGPS) criteria [13–15]. Post-operative mortality was defined as any death occurring within 30 days from surgery.

The following histopathological data were also evaluated: tumor dimension and grading (G), number of harvested lymph nodes, number of positive lymph nodes and resection margins (R) status. R status was defined as R1 when tumoral cells were found within 1 mm of the resection margin, according to the definition of the British Royal College of Pathologists[16].

In the ePD group, the evaluated margins included the anterior and posterior pancreatic surfaces, SMV groove, retroportal lamina (the closest margin to the superior mesenteric artery), common bile duct, and pancreatic stump. In the sLP group, the assessed margins were the posterior/retroperitoneal and anterior pancreatic surfaces as well as the pancreatic stump. The 8^th^ edition of the AJCC/UICC system was used for TNM staging[17].

Follow-up was scheduled according to oncologists’ recommendations and included physical examination, laboratory tests and computed tomography (CT) scan every 6 months. Magnetic resonance imaging (MRI) and/or whole-body positron emission tomography (PET) scan with 18-fluoro-2-desoxy-glucose (FDG) were performed in case of inconclusive CT findings. Recurrence was defined as local in case of tumor relapse along the superior mesenteric and/or celiac vessels, pancreatic remnant, pancreatic lodge or regional lymph-nodes, or as distant in case of relapse in any other site. Long-term outcomes included OS and DFS. OS was defined as the time from surgery to the last follow-up or death, while DFS was defined as the time from surgery to the detection of either local or distant tumor recurrence.

### Operative technique

The type of procedure (ePD or sLP) was planned preoperatively at the discretion of the operating surgeon and subsequently confirmed intraoperatively based on surgical findings. In all cases, lymph node stations were classified according to the Japanese nomenclature[18]. For patients undergoing ePD, pancreatic parenchyma transection was performed at the left border of the PV. Lymphadenectomy included stations #5, 6, 8a, 8p, 12b, 12c, 13a, 13b, 14a, 14b, 17a and 17b according to the indications proposed by ISGPS[19], with the addition of stations #9 and 11p. A Child reconstruction was performed in all cases, using the same jejunal loop to create a duct-to-mucosa pancreatico-jejunostomy, the hepaticojejunostomy and the gastrojejunostomy. The gastro-jejunal anastomosis was created at least 60 cm distal to the hepaticojejunostomy, in a side-to-side, antecolic manner[20].

In cases of sLP, pancreatic dissection was carried out up to the root of the GDA and splenectomy was routinely performed. Lymphadenectomy included stations #9, 10, 11p, 11d and 18 according to the indications proposed by ISGPS[19], with the addition of stations #8a and 8p. Therefore, in both procedures, lymph node dissection was performed along the CHA (#8a and 8p), celiac artery (CA) (#9) and the proximal splenic artery (SA) (#11p) due to the specific anatomical location of the tumor, in accordance with previous publications [6, 7].

Intraoperative frozen section analysis was routinely performed on the biliary and pancreatic transection margins to assess the need for further resection.

### Study outcomes

The primary endpoint of the present study was to compare the ePD and sLP cohorts in terms of long-term oncological outcomes, namely OS and DFS. The secondary endpoint was a further comparison between the two groups in terms of peri-operative outcomes.

### Statistical analysis

Continuous data were reported as median and quartile rank (QR), while all categorical variables were expressed as number and percentages. Student’s t tests, Mann–Whitney U tests, Fisher’s tests and χ^2^ test were used for the univariate analysis. A *p* value ≤ 0.05 was considered statistically significant for all the analyses performed. OS and DFS were calculated using Kaplan–Meier curves, and the multivariable analysis was performed using Cox proportional hazard model. Results were reported as odd ratio (OR) with 95% confidence intervals (CI). All tests were performed using SPSS version 25 for Windows (SPSS Inc., Chicago, IL, United States).

## Results

### Clinico-demographic characteristics of the study population and perioperative course (Table 1)

**Table 1:** Patient characteristics and perioperative outcomes according to surgical procedure.

	ePD (n = 18)	sLP (n = 28)	*p*
*Sex, n (%)*			
Male	6 (33.3)	14 (50)	0.26
Female	12 (66.7)	14 (50)	
Age (years), median* (QR)*	72 (66–77)	65 (59–73)	0.08
BMI*,* median* (QR)*	22 (21–24)	25 (23–28)	**0.02**
ASA score ≥ 3*, n (%)*	4 (22.2)	5 (17.9)	0.71
NAT, *n (%)*	8 (44.4)	3 (10.7)	**0.009**
Operative time (min), median* (QR)*	361 (327–407)	260 (231–289)	** < 0.0001**
Venous resection, *n (%)*	5 (27.8)	11 (42.3)	0.42
Clavien-Dindo ≥ 3, *n (%)*	5 (27.8)	3 (10.7)	0.14
*POPF, n (%)*			
No	14 (77.8)	17 (60.7)	0.1
BL	0 (0)	6 (21.4)	
B	3 (16.7)	5 (17.9)	
C	1 (5.5)	0 (0)	
PPH, *n (%)*	3 (16.7)	1 (3.6)	0.12
Reoperation, *n (%)*	1 (5.5)	1 (3.6)	0.75
LOS (days)*,* median* (QR)*	14 (10–26)	7 (6–9)	** < 0.0001**
In-hospital mortality, *n (%)*	1 (5.5)	0 (0)	0.2

ePD, extended pancreaticoduodenectomy; sLP, subtotal left pancreatectomy; BMI body mass index; ASA, American Society of Anesthesiologists; NAT neoadjuvant treatment; POPF, Post-operative pancreatic fistula; BL, biochemical leak; PPH, post-pancreatectomy haemorrhage; LOS, length of hospital stay.

Bold refers to statistically significant values.

**Fig. 1:**
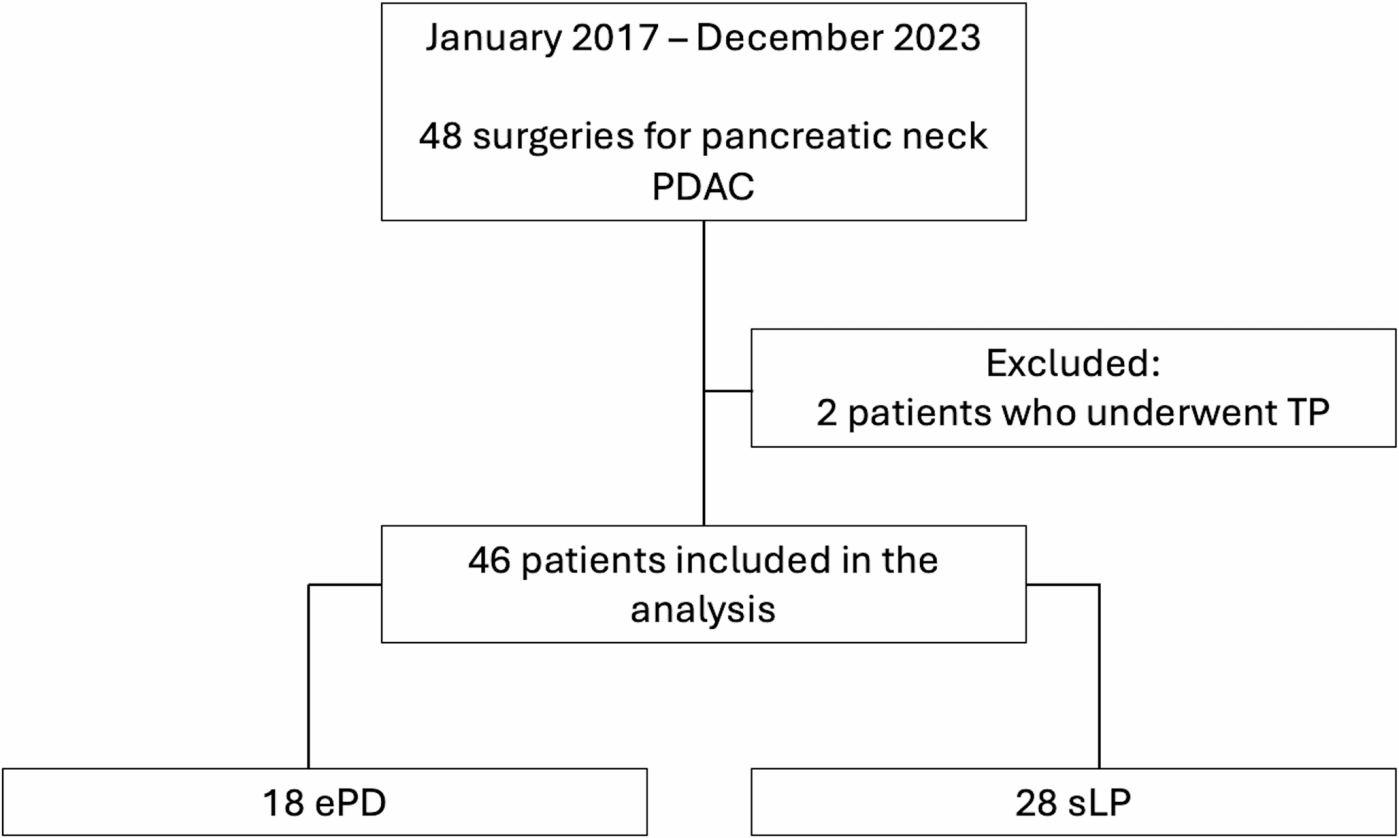
Flowchart showing patient inclusion and allocation.

From January 2017 to December 2023, 48 patients referred to the Pancreatic Surgery Unit of the Fondazione Policlinico Universitario Agostino Gemelli IRCCS of Rome for pancreatic neck PDAC. Two patients underwent TP and were therefore excluded from the analysis. The remaining 46 patients constituted the study cohort and were divided according to the type of surgical procedure performed: 18 patients (39.1%) underwent ePD and 28 (60.9%) underwent sLP (Fig. 1). No significant differences were observed in terms of median age and ASA score between the two groups. However, patients in the sLP group had a significantly higher BMI compared to those in the ePD group (*p* = 0.02).

NAT was administered in 11 (23.9%) patients with a significantly higher proportion in the ePD group (8–44.4% patients) compared to the sLP group (3 patients − 10.7%) (*p* = 0.009).

The median operative time was significantly longer for ePD (361 (327–407) minutes) in comparison to sLP (260 (231–289) minutes) (*p* < 0.0001). Venous resection was performed in 16 cases (34.8%): 5 (27.8%) in the ePD group and 11 (42.3%) in the sLP group (*p* = 0.42). In all cases of venous infiltration, a tangential resection of PV or PV/SMV was performed. Clavien-Dindo ≥ 3 complications occurred in the 17.4% (8 patients) of the whole population with no statistical difference between the two cohorts, although a higher rate was observed in the ePD group (5—27.8% vs 3—10.7% in the sLP group; *p* = 0.14). Rates of POPF, PPH, reoperation, and in-hospital mortality were similar between the two cohorts. However, length of hospital stay (LOS) was significantly longer after ePD compared to sLP (median 14 (10–26) days vs. 7 (6–9) days; *p* < 0.0001).

### Histopathological findings

At histopathological analysis (Table 2), no significant differences were observed between the ePD and sLP procedures in terms of T and N staging. Similarly, the distribution of positive lymph nodes at shared stations (#8a–p, #9, and #11p) was comparable between the two cohorts (*p* = 0.7). Of note, in the ePD group, positive lymph nodes were found in 8 patients (44.4%) at peripancreatic stations (#13–17), in 2 cases (11.1%) along the CHA (station #8a-p), in 1 patient (5.6%) at the CA (sation#9), and in 1 patient (5.6%) at the origin of the SA (sation #11p). In the sLP group lymph node involvement was observed in 12 patients (42.9%) at the peripancreatic station #18, in 3 patients (10.7%) along the CHA (#8a–p), in 2 patients (7.1%) at the CA (#9), and in 1 patient (3.6%) at the SA (#11p).

**Table 2:** Comparison of histopathological outcomes.

	ePD n = 18	sLP n = 28	*p*
*Grading, n (%)*			
G1	2 (11.1)	0 (0)	0.07
G2–3	16 (88.9)	28 (100)	
Tumor dimension (mm), median (QR)	21 (18–27)	25 (21–31)	0.2
R1, *n (%)*	5 (27.7)	6 (21.4)	0.62
*R1 site, n (%)*			
Anterior surface	0 (0)	0 (0)	**0.02**
Posterior surface	0 (0)	0 (0)	
Pancreatic stump	1 (5.5)	6 (21.4)	
SMV groove	4 (22.2)	0 (0)	
Retroportal lamina	0 (0)	0 (0)	
Common bile duct	0 (0)	–	
T stage ≥ 3,* n (%)*	0 (0)	5 (17.9)	0.06
N stage ≥ 1,* n (%)*	9 (50)	13 (46.4)	0.81
No. of harvested lymph nodes, median (QR)	16 (12–27)	15 (12–21)	0.5
No. of positive lymph nodes, median (QR)	1 (0–2)	1 (0–3)	0.7
*Site of positive nodes, station, n (%)*			
Suprapyloric lymph nodes #5	0 (0)	–	0.7*
Infrapyloric lymph nodes #6	0 (0)	–	
Common hepatic artery #8a-8p	2 (11.1)	3 (10.7)	
Celiac artery #9	1 (5.6)	2 (7.1)	
Splenic hilum #10	–	0 (0)	
Along the proximal splenic artery #11p	1 (5.6)	1 (3.6)	
Along the distal splenic artery #11d	–	0 (0)	
Bile duct and cystic duct#12b-c	0 (0)	–	
Peripancreatic #13–17	8 (44.4)	–	
Proxima superior mesenteric artery#14a-b	0 (0)	–	
Peripancreatic #18	–	12 (42.9)	

ePD, extendend pancreaticoduodenectomy; sLP, subtotal left pancreatectomy.

*p value is referred to the comparison between the two groups for the shared lymph node stations (#8a-p, #9, #11p).

Bold refers to statistically significant values.

Overall, 11 patients (23.9%) had an R1 resection, with no significant difference between the ePD and the sLP groups (5–27.7% and 6—21.4%, respectively; *p* = 0.62). However, the site of margin positivity significantly differed between the two cohorts (*p* = 0.02). A higher rate of pancreatic transection margin positivity was observed in the sLP cohort (6 cases − 21.4%) compared to the ePD group (1–5.5%), while a positivity of the SMV groove margin was more frequently noted in the ePD cohort (4–22.2%) in comparison to the sLP one (0) (*p* = 0.02). Notably, all six patients with a positive pancreatic margin had a negative intraoperative frozen section margin at the time of surgery.

### Oncological outcomes

A comparable number of patients in the ePD (14–77.8%) and sLP (26–92.9%) groups received adjuvant therapy (*p* = 0.14). Specifically, 8 (44.4%) patients in the ePD group and 15 (53.6%) in the sLP group received a gemcitabine/nab-paclitaxel regimen, while 6 (33.3%) ePD patients and 9 (32.1%) sLP patients underwent FOLFIRINOX chemotherapy.

Overall, tumor relapse occurred in 31 (67.4%) patients, with no statistically significant difference between the ePD (14–77.8%) and the sLP (17–63%) procedures (*p* = 0.29). Similarly, the site of recurrence (local vs distant) did not differ significantly between the two cohorts (*p* = 0.51). As for the pattern of local recurrence, in the ePD group, 2 patients (11.1%) showed involvement of the pancreatic remnant, while in another 2 cases (11.1%) recurrence was detected as solid tissue growth surrounding CHA and SMV. In the sLP group, 4 patients (14.3%) presented with recurrence at the pancreatic remnant, and 3 (10.7%) patients developed pathological tissue around CA. In terms of distant recurrence, 10 (55.6%) patients in the ePD cohort developed hepatic metastases. In the sLP cohort, 7 (25%) patients presented with hepatic metastases, while 3 (10.7%) patients developed peritoneal carcinomatosis.

The median follow-up was 26 (11–33) months and 31 (17–50) months in the ePD and sLP groups, respectively (*p* = 0.12) with no difference between the two cohorts in terms of mortality at the last follow-up (13–72.2% in the ePD and 13–48.1% in the sLP groups, respectively; *p* = 0.11). Oncological outcomes are summarized in Table 3.

**Table 3:** Oncological outcomes.

	ePD n = 18 (%)	sLP n = 28 (%)	*p*
Adjuvant chemotherapy, *n (%)*	14 (77.8)	26 (92.9)	0.14
Tumor recurrence, *n (%)*	14 (77.8)	17 (60.7)	0.29
*Site of recurrence, n (%)*			
Local	4 (22.2)	7 (25)	0.51
Distant	10 (55.6)	10 (35.7)	
Follow up, median (months)	26 (11–33)	31 (17–50)	0.12
Mortality at the last follow-up,* n (%)*	13 (72.2)	13 (46.4)	0.11

ePD, extendend pancreaticoduodenectomy; sLP, subtotal left pancreatectomy.

**Fig. 2:**
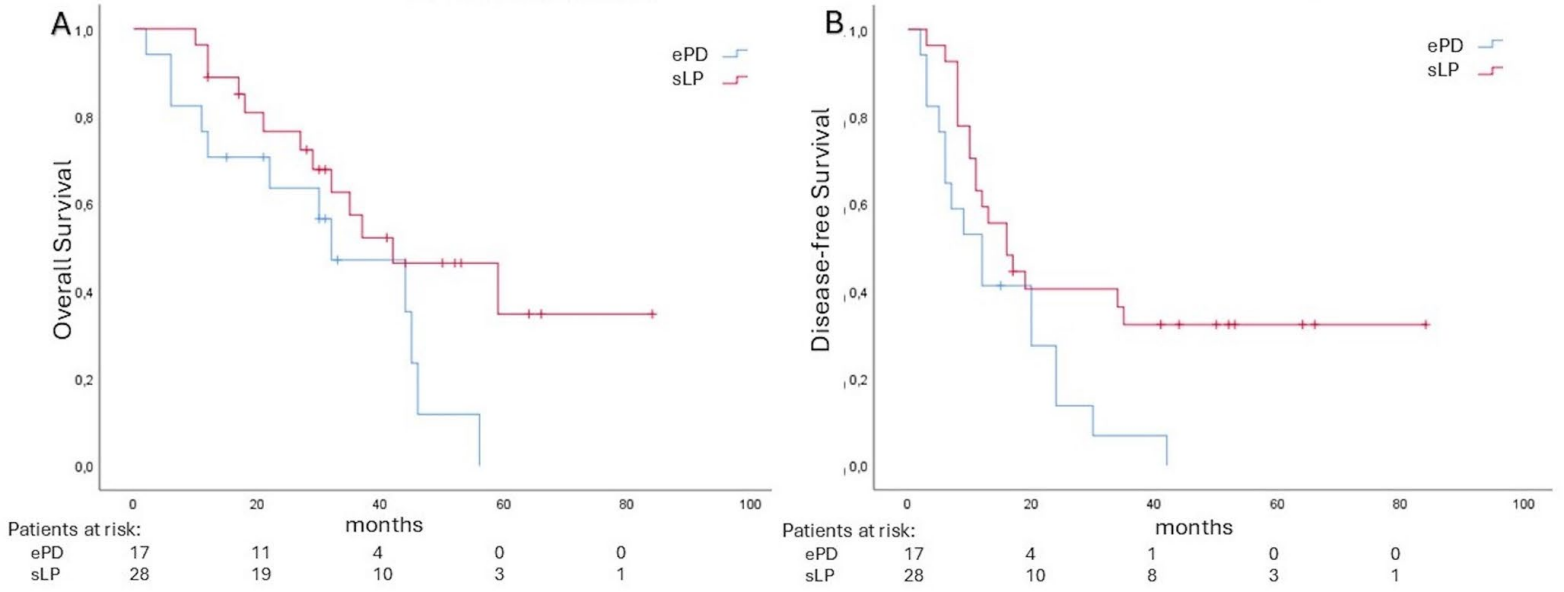
Kaplan–Meier curves comparing OS (**A**) and DFS (**B**) between ePD and sLP.

At the long-term outcomes evaluation, sLP was associated with a longer median OS (42 (19–65) months) compared to ePD (32 (14–50) months), although this difference did not reach the statistical significance (*p* = 0.06) (Fig. 2A). In contrast, a statistically significant advantage in DFS was observed for sLP (16 (9–22) months) compared to ePD (12 (5–18) months) (*p* = 0.03) (Fig. 2B).

At univariate analysis for OS (Table 4), only an ASA score > 3 (*p* < 0.0001) was significantly associated with a worse survival. For DFS, both an ASA score > 3 (*p* = 0.005) and ePD (*p* = 0.03) were negatively associated with the outcome (Table 4). These factors were also confirmed as independent prognostic indicators for worse DFS at the multivariate analysis (OR: 4.35, 95% CI 1.6–11.7; *p* = 0.004 for ASA score ≥ 3 and OR: 0.38, 95%CI 0.17–0.8; *p* = 0.009 for ePD).

**Table 4:** Univariate and multivariate analysis for OS and DFS.

Variable	OS		DFS			
	Univariate analysis		Univariate analysis		Multivariate analysis	
	OR (95% CI)	*p*	OR (95% CI)	*p*	OR (95% CI)	*p*
Sex	0.84 (0.4–1.8)	0.65	0.87 (0.44–1.7)	0.69		
Age > 65 years	2.2 (1–5)	0.06	1.47 (0.74–2.9)	0.27		
ASA ≥ 3	12.7 (3.85–41.9)	** < 0.0001**	3.51 (1.46–8.44)	**0.005**	4.35 (1.6–11.7)	**0.004**
ePD vs sLP	0.48 (0.2–1.1)	0.06	0.45 (0.23–0.89)	**0.03**	0.38 (0.17–0.8)	**0.009**
Tumor dimension ≥ 25 mm	1.2 (0.5–2.8)	0.68	1.1 (0.5–2.1)	0.87		
T3 vs T1-2	1.6 (0.46–5.48)	0.46	1.9 (0.73–5.14)	0.18		
N +	1.6 (0.73–3.5)	0.24	1.31 (0.68–2.55)	0.42		
R1 status	2.13 (0.95–4.78)	0.07	1.48 (0.69–3.18)	0.31		
Adjuvant therapy	0.97 (0.23–4.2)	0.97	0.87 (0.3–2.48)	0.79		

OS, overall survival; DFS, disease-free survival; ASA, America Society Anesthesiologists; ePD, extendend pancreaticoduodenectomy; sLP, subtotal left pancreatectomy.

Bold refers to statistically significant values.

## Discussion

The surgical approach to PDAC largely depend on tumor location, with PD and DP being the gold standard treatments for tumors of the pancreatic head and body-tail, respectively. Conversely, limited evidence is currently available in the literature regarding the optimal surgical approach for tumors located in the pancreatic neck. This is primarily due to the rarity of this tumor localization and the frequent detection of locally advanced lesions at initial diagnosis, given the proximity of this anatomical region to major vascular structures. In addition, limited and inconclusive data are currently present on the biological behavior of neck PDACs, particularly in terms of lymphatic drainage[4, 7, 8] . This inevitably contributes to further uncertainty in defining the most appropriate oncologic surgical approach. As a result, the techniques currently proposed range from ePD to sLP and TP, without conclusive evidence supporting one over the others[4, 5, 7, 8] .

With the aim of giving our contribution to this ongoing debate, we retrospectively analyzed the long-term outcomes of ePD and sLP for the treatment of neck PDACs, in order to evaluate the potential prognostic advantages of one procedure over the other. To accomplish this purpose, we retrospectively included all patients who underwent surgery for a pancreatic neck PDAC without involvement of the GDA.

One of the main current limitations in comparing and generalizing existing evidence lies in the inconsistent and often unclear definition of the pancreatic neck. Hirono et al.[7] defined the pancreatic neck as the pancreatic segment between the left border of the PV and the GDA, whereas the General Rules for the Study of Pancreatic Cancer[21] defined it as the portion of the pancreatic parenchyma anterior to the SMV and PV. In our study, neck PDACs were defined as those tumors arising in the 2-cm pancreatic segment between the right and left border of the PV/SMV, without infiltration of the GDA, similarly to the definition proposed by Imamura et al.[6]. The exclusion of cases with GDA involvement was specifically aimed to reduce potential biases in the selection of ePD as surgical procedure and to ensure maximal homogeneity in tumor location within the study cohort.

According to our results, no significant differences were observed in clinicodemographic characteristics between the study groups, although a significantly higher proportion of patients in the ePD group received NAT. Regarding perioperative outcomes, a shorter median operative time and LOS were documented in the sLP cohort. These advantages clearly depend on the different complexity of the two procedures, that make sLP easier to perform as compared to ePD. Histopathological analysis revealed comparable overall R1 resection rates between the two groups. However, sLP was associated with a higher incidence of positive pancreatic transection margins, whereas ePD more frequently showed positivity at the SMV groove margin. Of note, a high variability was observed in of lymph nodes metastasis distribution. In the sLP group, station #18—routinely not dissected during ePD—showed a 42.9% rate of positivity. Conversely, in the ePD group, stations #13 and #17—typically not dissected during sLP—were positive in 44.4% of cases. This variability highlights the ongoing debate surrounding the lymphatic drainage patterns of the pancreatic neck and their prognostic implications for resected patients. In this context, Nomura et al.[8] conducted a retrospective analysis of 35 patients with pancreatic neck PDACs to determine the optimal extent of lymph node dissection, applying the efficacy index previously introduced by Sasako et al. [22]. The authors reported metastatic involvement rates of up to 14% in stations #13 and #14, 9.5% in station #17, 17.1% in station #8, and 28.6% in station #11p. Station #11p had the highest efficacy index, followed by stations #14 and #13. Notably, no metastases were observed in station #18. In our cohort of ePDs, stations #13 and #17 were the most frequently involved, followed by stations #8 and #11p, in line with the report of Nomura et al. [8]. Conversely, in the sLP cohort, we observed a metastasis rate up to 42.9% at station #18, followed by station #8, #9 and #11p. The high incidence of lymph node metastases in peripancreatic stations of the pancreatic head, not routinely dissected during sLP, and along the left inferior margin of pancreas, not dissected during ePD, suggest a potential risk of oncological undertreatment when either ePD or sLP is adopted for the surgical treatment of pancreatic neck PDACs. This paves the way to the hypothesis that TP may represent the most appropriate treatment strategy to ensure an oncologically correct procedure for pancreatic neck PDACs. This consideration is further supported by the finding that up to 21% of sLP patients had a positive pancreatic transection margin—an issue that would be inherently resolved by removing the entire pancreatic gland. In this regard, positivity of the pancreatic transection margin in the sLP cohort was only identified postoperatively, whereas intraoperative frozen section analysis had shown negative margins. This precluded the possibility of performing conversion surgeries, such as TP, which might have ensured greater oncological radicality.

However, the indication to TP has been historically limited due to its association with higher morbidity, mortality rates and significant metabolic consequences compared to other pancreatic surgical procedures[23]. Nevertheless, recent advancements in surgical techniques and perioperative management have led to rates of major morbidity and mortality after TP that are comparable to those of PD[24, 25], with no significant differences in postoperative quality of life (QoL)[26]. Despite this progress, current evidence in the literature regarding the role of TP in the treatment of pancreatic neck PDACs remains limited. Only Zengh et al.[10] and Rompen et al.[27] have conducted comparative analyses of PD, LP, and TP for the treatment of pancreatic neck PDAC, using data from the Surveillance, Epidemiology, and End Results (SEER) database and the National Cancer Database (NCDB), respectively. Although neither study demonstrated a clear oncologic advantage of TP over PD or LP, several significant limitations must be acknowledged.

First, both databases lacked key oncologic data such as margin status, detailed information on neoadjuvant and adjuvant treatment regimens, and the timing of these therapies. Second, no standardized definition of the pancreatic neck was adopted, and surgical decisions may have been influenced by the tumor exact location—for instance, involvement of the GDA—or by potential infiltration of surrounding vascular structures. Third, in both studies, the TP cohorts showed significantly higher rates of lymph node positivity compared to the PD and LP cohorts, which could have confounded survival outcomes. These limitations inevitably affect the reliability of the findings and underscore the need for further, well-designed studies to properly assess the role of TP in the treatment of pancreatic neck PDAC.

Regarding long-term outcomes, no significant difference in OS was observed between the two procedures, although a slight advantage was noted for sLP over ePD. DFS, however, was significantly better in the sLP group, and sLP emerged as an independent prognostic factor for prolonged DFS compared to ePD. These findings should be interpreted with caution, especially in light of the differences in NAT rates and post-operative complications between the two procedures. A higher proportion of patients in the ePD group received NAT compared to those in the sLP group, potentially indicating more advanced or aggressive disease, which may have negatively influenced long-term outcomes. Similarly, although not reaching the statistical significance, ePD was associated with more than twice the rate of Clavien-Dindo grade ≥ 3 postoperative complications. The detrimental impact of major postoperative adverse events on oncologic prognosis has been extensively documented in the literature[28–31].

This study has several limitations. The retrospective design and the small sample size of the two cohorts—although comparable to other reports currently available in the literature—represent the main drawbacks. These factors inevitably resulted in two heterogeneous comparative groups, particularly regarding key prognostic variables such as the rate of NAT. Such imbalance between the two cohorts may have introduced a potential bias affecting the results, particularly with respect to OS, recurrence patterns, and postoperative complication rates. Similarly, the limited number of patients may have affected the statistical power in evaluating independent prognostic factors, as demonstrated by the absence of a significant correlation between R status or N status and long-term outcomes, despite their well-established prognostic value in the literature[32]. Conversely, to the best of our knowledge, this is the first study to specifically compare the two proposed surgical strategies—ePD and sLP—for the treatment of pancreatic neck PDAC, based on a predefined anatomical definition of the pancreatic neck region.

In conclusion, based on our findings, both ePD and sLP are likely to be inadequate to ensure an oncologically correct procedure for neck PDAC, particularly given the high incidence of lymph node metastases in station #18 (not resected during ePD) and stations #13 and #17 (not resected during sLP). These results support the hypothesis that TP may represent a more oncologically appropriate treatment option for tumors located in the pancreatic neck. However, this potential oncological advantage must be carefully balanced against the metabolic derangements and QoL impairments associated with the complete loss of pancreatic function. Nonetheless, there is a clear need for further and more extensive comparative studies—ideally multicenter, prospective investigations adopting a standardized anatomical definition of the pancreatic neck—in order to better assess the prognostic impact of the different surgical approaches in this specific subset of patients.
